# Experiences of an Online Treatment for Adolescents With Nonsuicidal Self-injury and Their Caregivers: Qualitative Study

**DOI:** 10.2196/17910

**Published:** 2021-07-23

**Authors:** Olivia Simonsson, Hedvig Engberg, Johan Bjureberg, Brjánn Ljótsson, Julia Stensils, Hanna Sahlin, Clara Hellner

**Affiliations:** 1 Centre for Psychiatry Research Department of Clinical Neuroscience Karolinska Institutet, & Stockholm Health Care Services, Region Stockholm Stockholm Sweden; 2 Department of Women's and Children's Health Karolinska Institutet Stockholm Sweden; 3 Medical Unit of Gynecology and Reproductive Medicine Karolinska University Hospital Stockholm Sweden; 4 Department of Clinical Neuroscience Division of Psychology Karolinska Institutet Stockholm Sweden

**Keywords:** nonsuicidal self-injury, self-injurious behavior, online treatment, internet, digital health, emotion regulation, emotion regulation individual therapy for adolescents, adolescent, qualitative, experience

## Abstract

**Background:**

Nonsuicidal self-injury (NSSI) is common in adolescence and is associated with several adverse outcomes. Despite this, few established treatment options exist. Online treatment seems promising for several conditions; however, knowledge on NSSI is scarce. It is important to explore how online treatment for NSSI is experienced to improve such interventions and learn more about factors that are important in the treatment of adolescents with NSSI.

**Objective:**

This study aims to explore the experiences of a novel online treatment for adolescents with NSSI and their caregivers.

**Methods:**

A qualitative study using thematic analysis was conducted through semistructured interviews with 9 adolescents and 11 caregivers at treatment termination or at the 6-month follow-up of the online emotion regulation individual therapy for adolescents.

**Results:**

A total of 3 overarching themes were identified. The theme *support can come in different shapes* showed how support could be attained through both interaction with the therapist as well as through the format itself (such as through the fictional characters in the material and the mobile app). Caregivers found it helpful to have their own online course, and adolescents accepted their involvement. The theme *self-responsibility can be empowering as well as distressing* showed that self-responsibility was highly appreciated (such as deciding when and how to engage in treatment) but also challenging; it caused occasional distress for some. The theme *acquiring new skills and treatment effects* showed the advantages and challenges of learning several different emotion regulation skills and that decreased emotion regulation difficulties were important treatment outcomes for adolescents. In addition, several different skills seemed to facilitate emotion regulation, and having access to such skills could hinder NSSI.

**Conclusions:**

Online emotion regulation individual therapy for adolescents seems to offer an accepted way to deliver family interventions for this target group; facilitate skills training with several means of support, including support from both the mobile app and the therapist; contribute to decreasing emotion regulation difficulties and teaching skills that could hinder NSSI; and cause (in some individuals) distress because of the self-responsibility that is inherent to online formats, which needs to be addressed.

## Introduction

### Background

Nonsuicidal self-injury (NSSI) is common in adolescence and associated with several long-term risks. NSSI refers to the “direct and deliberate destruction of body tissue in the absence of any observable intent to die” [1]. NSSI is a symptom of borderline personality disorder but is also present in the absence of borderline personality disorder [2,3], together with several other disorders [4,5], and in nonclinical populations [6]. NSSI is common, especially in midadolescence [7], with a pooled prevalence of approximately 17% [8]. In fact, the prevalence rate seems to have increased in recent years [9,10]. NSSI in adolescence is worrying, as it is associated with an increased risk of incidence of other psychopathology in youth [11] and several adverse outcomes in adulthood [12-14]. Even though there is an overlap between NSSI and suicide attempts [4,15-17], there are some key differences between the two, concerning intention, repetition, and medical lethality [1,17-19]. In addition, NSSI is one of the strongest risk factors for future suicide attempts [20], and cessation of self-injury in adolescence can potentially decrease the risk of future suicidal behavior [21].

Therefore, NSSI is serious and requires urgent treatment. Treatments that target NSSI specifically are needed because NSSI is a transdiagnostic phenomenon [2-5], and targeting NSSI can potentially prevent suicides [21,22]. Focusing on the maintenance factors of NSSI in treatment could be useful; the emotion regulation function (ie, that one engages in NSSI to decrease or escape aversive emotions) has been identified as the most common function of NSSI [23], and treatment for NSSI that targets emotion regulation difficulties seems to have beneficial effects on other health outcomes as well [24]. At present, no well-established treatment is available for treating NSSI [25-29], but dialectical behavior therapy (DBT) is probably efficacious for adolescents [25]. Nevertheless, challenges with accessibility and costs connected to DBT have been highlighted and briefer interventions have been called for [22].

Our research group has previously developed a brief emotion regulation individual therapy for adolescents (ERITA) [30] derived from emotion regulation group therapy (ERGT) [24,31] drawing from the principles of DBT and acceptance and commitment therapy. The aim of ERITA and ERGT is to decrease NSSI through learning and using new skills to regulate emotions. In contrast to ERGT, ERITA is a 12-week individual therapy that is specifically for adolescents and includes a parallel online course for caregivers. As ERITA seems promising [30], and as online treatment has several advantages, such as (1) the possibility of frequent contact with the therapist [32], (2) seems effective for several conditions among adolescents [33], and (3) can facilitate help-seeking as fear of stigmatization can be a barrier [34] and individuals with perceived stigmatized problems may prefer online treatment [35], our research group has further adapted ERITA [30] to an online version (ie, online ERITA). The quantitative results from the pilot trial indicate that online ERITA seems acceptable, feasible, and useful [36]. Online ERITA was, in a recent systematic review, the only online treatment tested for adolescents with NSSI [37].

Consequently, as research on online treatment for adolescents with NSSI is scarce, our knowledge of the experience of online treatment is limited [38]. Collaboration with patients has been suggested to be important when developing novel interventions for self-injury [29]. One way to engage patients in the development of interventions is through qualitative research methods. Qualitative evaluations of interventions allow for individual experiences, positive and negative feedback, and could together with quantitative evaluations contribute to a richer understanding of an intervention [38]. Regarding adolescents with self-injury, there is only 1 previous qualitative study investigating the experience of online interventions, specifically the experience of a mobile app as an adjunct to face-to-face treatment [39]. To our knowledge, no study has investigated the experiences of online treatment for adolescents with NSSI nor have the experiences of caregivers been explored. Involving caregivers in treatment for self-injury has been suggested [40], and increased knowledge of the experience of such involvement, also from the caregivers’ perspective, could help to further develop successful treatment for adolescents with NSSI. Overall, exploring individual experiences of novel treatments can potentially help improve existing treatments as well as increase our understanding of factors that are important in the treatment of adolescents with NSSI.

### Objective

This study aims to explore the experiences of online ERITA for those with NSSI and their caregivers.

## Methods

### Overview

This qualitative study was part of a pilot trial of online ERITA [36]. This study was approved by the regional ethical review board of Sweden in November 2015 (reference number: 2015/1895-31/5). Furthermore, it is presented according to the Consolidated Criteria for Reporting Qualitative Research standards [41] for reporting qualitative research.

### Participants

Families who had participated in the quantitative part of the pilot trial of online ERITA [36] were the population of interest. Inclusion criteria for adolescents in the pilot trial [36] were as follows: (1) being 13-17 years old; (2) fulfilling criteria for the proposed NSSI disorder [42]; (3) having engaged in ≥1 NSSI episode during the past month at the preassessment; (4) having stability of psychotropic medications (if any) for at least 2 months; and (5) having at least one caregiver committed to participate in the caregiver course. Exclusion criteria for adolescents in the pilot trial [36] were as follows: (1) severe suicidal ideation; (2) diagnosis of psychotic or bipolar I disorder, or substance dependence; (3) ongoing dialectical behavioral or mentalization-based therapy; and (4) insufficient understanding of the Swedish language. To participate in the qualitative part of the pilot trial, participants had to accept participation in interviews and terminate treatment before May 14, 2017.

Potentially eligible families were informed and prompted to participate in the interviews, either when meeting their therapist at posttreatment follow-up or by telephone. From the 17 eligible families, families were continuously selected for interviews as the data collection proceeded based on maximum variation sampling [43]; the target was to reach variability in the sample regarding age, gender, and past month NSSI frequency before and after treatment. One family was selected for interviews but later declined. 

**Figure 1:**
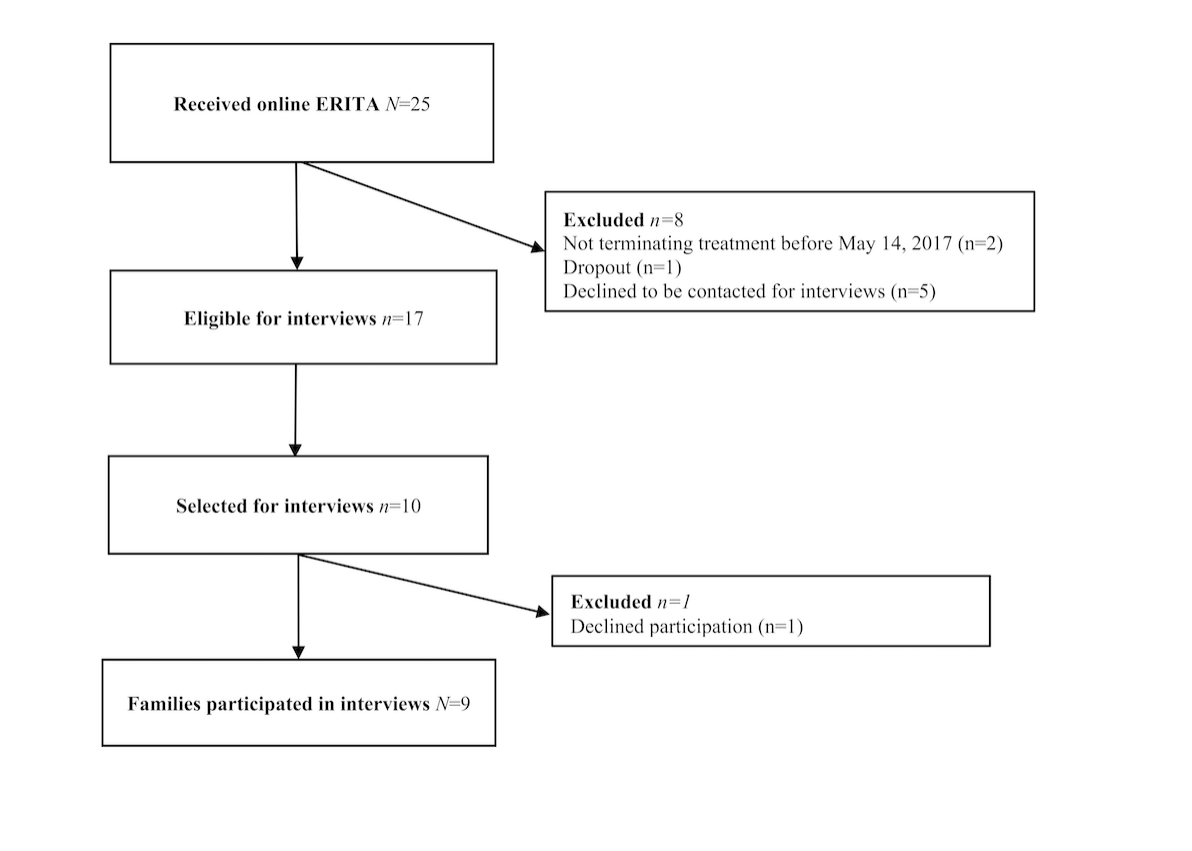
Flowchart of the participating families. ERITA: emotion regulation individual therapy for adolescents.

Information power [44] was assessed continuously by analyzing the amount of new information from the interviews and the quality of data. From previous research, 6 to 12 interviews have shown to capture all themes and concepts needed to answer the research question [44-47]. We expected that more than 6 interviews per group (ie, separating adolescents and caregivers) were needed based on the variability within the adolescent group and the scope of the study. Conversely, sample specificity was good in relation to the research question. After interviews with 9 families (ie, 9 adolescents and 11 caregivers), there was redundancy in interview data; therefore, it was deemed that a data saturation point was reached [48]. The participant flow is shown in Figure 1.

In total, 9 adolescents and 11 caregivers participated (ie, 20 interviews in total); 7 families completed the interview in conjunction with the posttreatment follow-up, and 2 families completed the interview in conjunction with the 6-month follow-up. Participating adolescents were 14-17 years old (median 16; IQR 16-17), and 6 adolescents identified as female and 3 adolescents as nonbinary. Before the initiation of the treatment, past month NSSI frequency varied between 3 and 22 (median 10; IQR 9-15), and after the treatment, the past month NSSI frequency varied between 0 and 14 (median 2; IQR 1-8). All adolescents had completed all the 11 modules in the treatment, and all caregivers had completed all 6 modules in the caregiver course. Of the participating caregivers, 9 were mothers and 2 were fathers. Their ages ranged from 43-55 years (median 50; IQR 46-53).

### Intervention

#### Overview

Online ERITA is developed by a diverse group, with experts in both online and self-injury treatment, in close collaboration with the developers of ERGT, and with help from a user experience design consultant. Furthermore, the content and online interface has been additionally reviewed by clinicians working with adolescent self-injury and patient representatives from an association specifically for self-injury.

**Figure 2:**
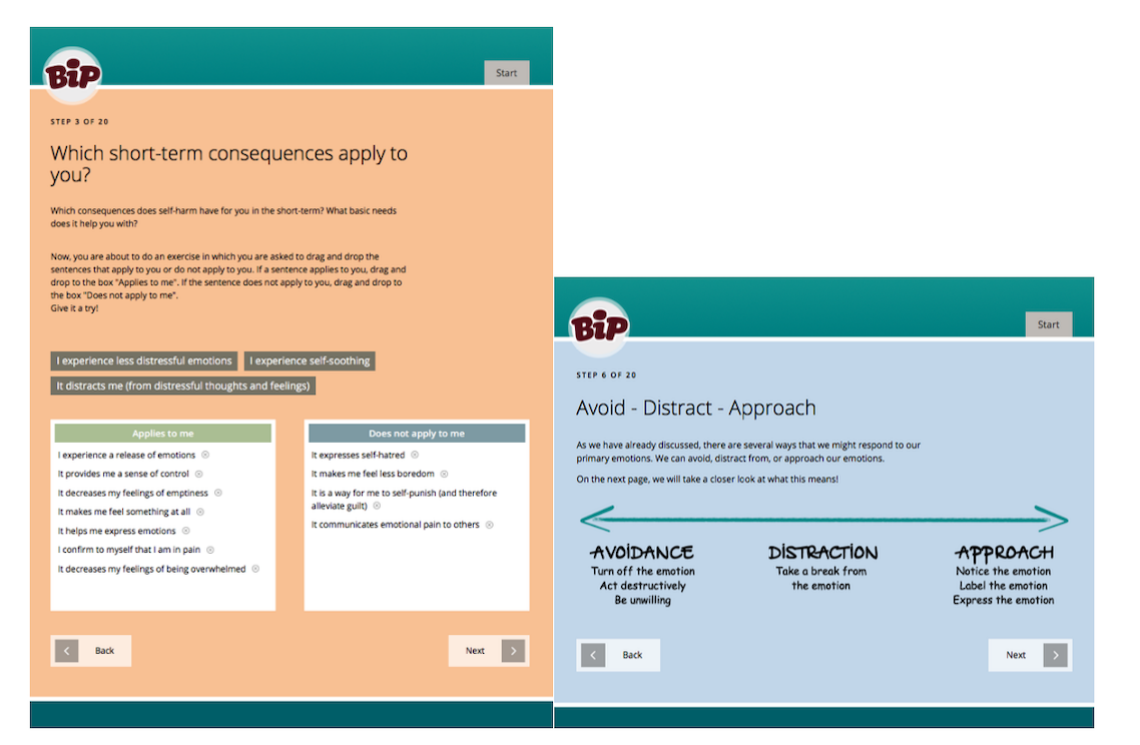
Screenshots of interactive worksheets from online emotion regulation individual therapy for adolescents [36].

**Figure 3:**
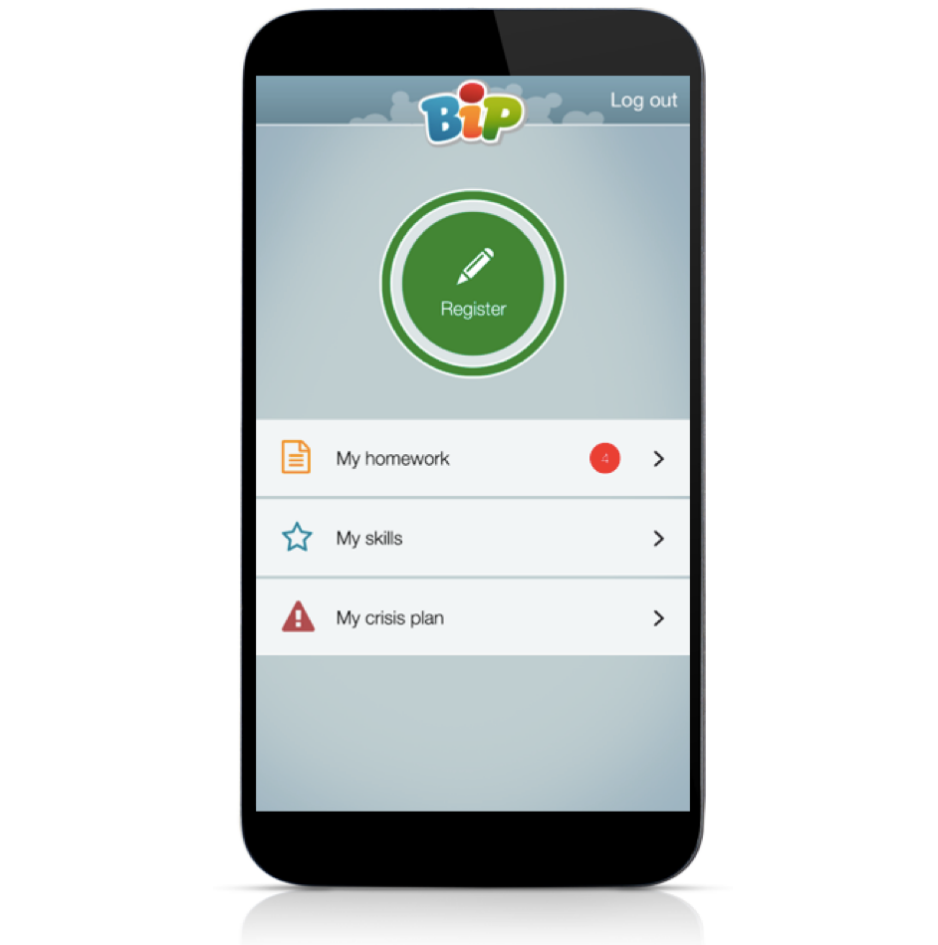
Screenshot of the mobile app from online emotion regulation individual therapy for adolescents [36].

The aim of ERITA is to decrease NSSI through learning and using new skills to regulate emotions. The treatment includes skills training in emotional awareness, acceptance, impulse control, validation, and valued direction. Online ERITA comprises 11 modules delivered over 12 weeks. In each module, participants read texts (ie, both psychoeducation and examples from how fictive characters practiced skills), listen to audio files, watch short films, and get assignments for the upcoming week. In addition, the adolescents have access to a supplementary mobile app where they can (1) register NSSI acts or impulses, (2) register and engage in their skills practice, and (3) access their individual crisis list. Screenshots of the online treatment and mobile app are presented in Figure 2 and Figure 3, respectively.

Moreover, online ERITA includes a parallel online course for the caregivers, which consists of 6 modules, with the aim to teach the caregivers emotion regulation strategies to better support and understand adolescents. The caregiver course includes skills training in validation, emotional awareness, and behavioral activation. The caregivers also have access to the module texts from the adolescent’s treatment, to learn what the adolescent is taught and encouraged to assist in their skills practice. The family meets their assigned online therapist for a face-to-face assessment before starting the treatment. During the treatment, both adolescents and caregivers have regular contact with the therapist on the online platform as well as by telephone if needed. In the secure online platform, the therapist has access to all interactions by adolescents and caregivers with the online system (eg, answered worksheets, registrations, and reflections on content, video, or audio) and data from the mobile app from adolescents (eg, daily registrations on destructive behavior and number of skills training sessions). The information is used by the therapist to assist in skills training, explain content if needed, monitor symptoms, and contact the family immediately in case any information indicates that the safety or well-being of adolescents is at risk.

#### Participant Safety and Confidentiality

Both adolescents and caregivers received information on how confidentiality would be handled in case any information indicated that the safety or well-being of adolescents was at risk (ie, contacting adolescents and/or caregivers) before consenting, and this was also discussed when developing the individual crisis list. Furthermore, the online platform was on a secure server, and log-in required 2-factor authentication (ie, both password and mobile code) for both the therapist and the participants. The mobile app was password-protected and locked to 1 phone. Adolescents were automatically logged out of the mobile app in case of inactivity, and data from the mobile app were saved on a secure server. The mobile app was designed to be discrete; the mobile app icon is neutral and cannot be associated with mental health. Moreover, participants could have initiated or did initiate treatment contacts outside of online ERITA based on their needs.

### Data Collection

Interviews were conducted between February and September 2017. The interviewers were 2 female psychology master students (OS and JS) educated in the theory and method of online ERITA, but they had no prior relation to the participants before the interviews. The interviews were conducted either at a child and adolescent mental health clinic (9 interviews), in the home of the families (6 interviews), or over telephone (5 interviews).

The interviews were conducted with the adolescents and caregivers in separate rooms, without nonparticipants. Before starting the interviews, the participants were informed about the purpose of the study; the occupation and experience of the interviewer; their right to terminate at any time; and how the material would be processed, stored, anonymized, and presented. Written informed consent (ie, caregivers and adolescents aged ≥15 years provided own written consent, and caregivers provided written consent on behalf of adolescents aged ≤14 years, according to Swedish law) was obtained before the interviews started. The duration of the interviews ranged from 13-41 minutes. To decrease the potential risk of the negative impact from talking about mental health issues and experiences of treatment, participants could choose the location of the interview, and all participants were asked before and after the interview if the situation induced distress. Interviewers were prepared to assist in case of reported or observed adverse events. However, no adverse events were reported before, during, or after the interviews.

Semistructured interviews were chosen based on the research questions and allowed for diverse experiences [49]. A total of 2 interview guides, 1 for the adolescents and 1 for the caregivers (Multimedia Appendices 1 and 2), were developed based on previous research (ie, on structure [49] and content [25,26,37,40]) and clinical experience and reviewed by the authors. The interview guide was pilot-tested on the first participant and evaluated with the supervisor (HE); no changes were deemed necessary. All interviews were audio recorded and later transcribed verbatim by the interviewers. The audio recording was erased immediately after the completion of the transcription.

### Data Analysis

Thematic analysis was used for data analysis, according to the steps recommended by Braun and Clarke [50]. Data analysis included a constant movement back and forth between the steps described below. In the first step, all transcripts were reread to gain familiarity with the data. In the second step, initial codes were generated; OS and JS coded 5 interviews together*,* and HE reviewed the coding as a verification step. After the verification step, OS coded the rest of the interviews and consulted HE throughout the process. All codes were reviewed several times by HE and OS. Examples of codes are listed in Table 1. In the third step, possible themes were investigated. Codes were clustered into categories and possible themes, and OS and HE reviewed different clustering opportunities and looked for negative cases. Data were split between adolescents and caregivers and compared in terms of both content and structure. In the fourth step, the themes were reviewed until they were deemed exclusive and fit the material. In the final step, the themes were labeled and the materials belonging to each theme were summarized. Discussions were ongoing in the research group throughout the process, and the final results were approved by all authors. Original quotes from the transcripts were chosen to show the relationship between the data and themes. For the qualitative analysis, the data program Open Code version 4.03 was used [51].

**Table 1:** Illustration of the resulting model.

Overarching themes and subthemes			Example of codes
**Support can come in different shapes**			
	Support from the therapist despite distance	Free in what one could say to the therapist Lonelier without the therapist Therapist cared	
	Finding support within the family	Work separately Caregivers more aware now Better communication	
	Finding support in the format	Could relate to the fictional characters Good to know you are not alone The app contributes with control	
**Self-responsibility can be empowering as well as distressing**			
	Flexibility and empowerment	Treatment was no big deal According to your needs Helped myself	
	Distress as a consequence of treatment	Hard to manage to support Pressure of constant accessibility Guilt when not following the treatment	
**Acquiring new skills and treatment effects**			
	Learning and using new skills	Use the skill that works in the situation Difficult to manage during emotional distress Too much to think about	
	Benefits in everyday life	Increased awareness Can use skills in everyday life Dare to express emotions	

## Results

A total of 3 overarching themes were identified. Each theme, their respective subthemes, and examples of codes in the themes are presented in Table 1.

### Support Can Come in Different Shapes

#### Overview

The overarching theme *support can come in different shapes* describes the diversity of how support can be attained, as defined by the separate subthemes. Although adolescents experienced support from several sources, caregivers focused more on support from the therapist.

#### Support From the Therapist Despite Distance

There was consensus that therapist support was an essential part of the treatment, among both adolescents and caregivers. The therapist was perceived as available, caring, supportive, personal, helpful, and pedagogical:

You could just ask anything, also questions that I thought were stupid, but still. Otherwise [without the therapist] it would have felt lonelier, as if you were just doing it by yourself, like nobody cared. Now there was someone who was there that wrote to you, after all. You got the response quickly when you sent a message, it felt good.

Adolescent #5

In addition, caregivers stressed the need for an initial face-to-face meeting to build trust. The initial meeting was also important as it decreased some caregivers’ worries about a somewhat lower level of caretaking in the online format compared with face-to-face treatment. Several caregivers wished for a face-to-face meeting halfway through the treatment period to assure the therapist was still there and to discuss problems that had emerged as treatment had progressed. According to most caregivers, the therapist was crucial—it felt as if somebody would take them on and wanted to help them:

It has been very important to have the therapist there, so you don’t feel that this is just digital. You need to have a real person there. The therapist has been very generous with the amount of contact. Both me and my adolescent have felt that the therapist aspect has been very important.

Caregiver #11

Adolescents appreciated the online communication with the therapist, and many preferred it to talking face-to-face. Nevertheless, telephone calls were appreciated as problem solving went quicker and the therapist could explain the treatment material that was hard to understand. For adolescents, the physical distance to the therapist seemed to increase their willingness to share sensitive personal information:

I simply find it easier to write, to get more time to think about exactly how to formulate myself...it can be easier to say things I don’t like to say aloud.

Adolescent #5

#### Finding Support Within the Family

Adolescents experienced the involvement of caregivers as reasonable and relevant, and some saw the advantages of caregivers getting their own support. Caregivers’ awareness of what the adolescent was going through (ie, through reading the adolescent’s material) facilitated communication and increased support. However, a few families expressed that they did not talk among each other about what they were going through:

They are more aware of what I do, that is, what assignments I get. And they can solve situations, kind of. But we haven’t talked so much about it. We have rather done it individually.... But now they have helped themselves and I have helped myself and it is like, I have not had to push them away in the same way [as before].

Adolescent #3

Caregivers were appreciative that they were more involved in online ERITA compared with previous treatments. Some requested more interactive parts with the adolescent for a natural starting point to communicate around the treatment material. Both adolescents and caregivers stated a better and more supportive relationship exemplified as talking more, having a common language, being more honest with each other, and having fewer arguments:

It feels like we’ve come closer. Before, she was in her room and you didn’t get any contact. And it may well happen that she does that now too, but it’s not that often. She is seeking much more social contact.

Caregiver #8

#### Finding Support in the Format

Adolescents found the treatment format and content to be supportive. The fictional characters in the modules were perceived as relatable and created a sense of normalization for some:

There were always examples of four people, and I could always recognize myself in at least one of them. Sometimes you might not recognize yourself up to a hundred percent, but there was always something you could recognize that made you feel less alone and “all right, it’s not just me who has this problem.”

Adolescent #4

Moreover, adolescents who used the supplementary mobile app appreciated it. Practicing skills in the mobile app and getting suggestions of what to do made the treatment more present and supported everyday skills training. The reasons mentioned for not using the mobile app were not experiencing the need or technical issues (eg, not being able to log in):

I think the app was the best. I probably logged in to it more times than I really needed. More as a reminder to me.... Sometimes it was hard to remember what to do in a situation when I was feeling very, very bad. If I logged in to the app, I had more control. Otherwise, I find it very difficult to come up with it [strategies] myself.

Adolescent #9

### Self-Responsibility Can Be Empowering as Well as Distressing

#### Overview

The overarching theme *self-responsibility can be empowering as well as distressing* describes the positive and negative aspects of the perceived self-responsibility of initiating and following through with the treatment. The subthemes reflect the positive aspects, such as you could engage in treatment according to your preferences and attribute accomplishments to your own ability, and the possible negative aspects, such as feeling distressed and insecure.

#### Flexibility and Empowerment

Adolescents and caregivers appreciated that the material was always accessible and that one could engage in treatment wherever it felt comfortable. Furthermore, flexibility in how to engage with the material (eg, text, audio files, videos, and mobile app) and freedom to write to the therapist whenever you have a question were similarly mentioned as positive consequences of self-responsibility:

It didn’t become such a big deal as to go somewhere, meet someone and talk. Rather it was more like sitting at home on the couch, just filling out some questions.... You could get a question, feeling it was tough, get up and go and grab a sandwich, talk it through with a parent and then go back and work on an answer.

Adolescent #1

Other positive aspects of self-responsibility, mentioned by adolescents, were connected to empowerment; feeling that you have the treatment to yourself and help yourself (rather than just receive help) and to not burden anyone else:

It didn’t feel like I was troubling anyone else in any way with my mental health problems. It was just me trying to get better.

Adolescent #3

#### Distress as a Consequence of Treatment

Engaging in and experiencing the responsibility of the treatment was, at times, connected to aversive emotions and unhelpful behaviors. The sense of failing assignments and expectations was associated with elevated levels of shame and guilt and decreased self-confidence. Being aware of one’s problems (ie, NSSI and difficulties in regulating emotions) was difficult for some, and the treatment was a reminder of those problems. When adolescents felt that they did not meet expectations, some ended up procrastinating and avoiding treatment; still, it was hard to mentally let go of the treatment. However, such aversive emotions were also described by some as manageable, transient, and acceptable because the treatment was important:

When I felt that I would not be able to do as many homework assignments as I wanted to do, then I did nothing instead, and finally I felt more stressed because I did nothing.... It wasn’t just a meeting; it was all the time. And sometimes it felt good because then it was like, you helped yourself all the time and you got help all the time. But at the same time, it was really hard because a meeting with a psychologist is usually really exhausting. And now it was like it went on all the time, that you had to think about all the stuff and all the questions, so it was just as hard, but good.

Adolescent #3

Both adolescents and caregivers experienced insecurities about what was *right* and *enough* in terms of how to answer homework assignments and what was expected of them. Rarely did adolescents or caregivers mention such concerns to their therapists:

One problem was that I had a hard time formulating answers to the questions in the module, so I don’t really know if I...came through with all my thoughts to the therapist. I thought the messages worked well, but I was always unsure how much I should write in the questions in modules-How deep should I go?

Adolescent #1

Some caregivers expressed doubts about their own capabilities, especially trusting the adolescent with responsibility and encouraging independence. The responsibility to motivate and remind adolescents was challenging for some:

Some days we felt that the adolescent did not manage to do the assignments and she received quite a lot of reminders, and we got a lot of reminders to remind her and so on. And it was very difficult to push her.

Caregiver #5

### Acquiring New Skills and Treatment Effects

#### Overview

The overarching theme *acquiring new skills and treatment effects* describes the outcome of participating in online ERITA. The subthemes reflect both learning skills and the positive effects of using such skills.

#### Learning and Using New Skills

Adolescents mentioned several different skills as helpful. The particular skills that were most helpful differed between adolescents and over time. Learning how to combine skills during this delimited time period was challenging for some, especially how to use the skill set in challenging situations:

I thought it was a little difficult in the moment, when I was feeling bad, to try to focus on all these different steps because there were so many different things you can do. So, it got a little confusing. I couldn’t really use the whole thing as a package.

Adolescent #6

Regarding the less helpful skills, some mentioned mindfulness as unimportant. Others were unable to specify in retrospect if anything was less helpful. In cases where participants perceived specific content as nonrelevant, they did not indicate that this content had had an overall negative impact on the experience of the treatment. Moreover, some experienced that some skills (particularly distraction skills) were insufficient, not solving problems in life:

It felt more like all skills became like distractions to me, more like I was postponing problems, instead of...I mean, I understand that it is a way of handling one’s problems, and that it is perhaps better than just ignoring it, but it didn’t feel like it helped a lot at the time.

Adolescent #2

Caregivers appreciated the fact that they received their own skills training and discovered that they could benefit from the same skills as adolescents. Learning about primary and secondary emotions and validation was perceived as particularly useful. Validation was defined as a eureka moment and a breaking point. Furthermore, the caregivers did not perceive any treatment content as less helpful, and several caregivers made the spontaneous reflection that everyone should learn these types of skills.

#### Benefits in Everyday Life

The improvements reported were both overt behavioral changes as well as changes in attitude toward internal experiences. Specifically, the improvements expressed by adolescents were increased emotional awareness and acceptance, courage to be who you are, skillful communication of emotions, and reaching out for help before making the situation worse:

I have become very much more aware of how I really work and how emotions and thoughts work and...I don’t know, just a lot of knowledge. It’s been amazing! And can help friends a little bit too, so it’s cool.

Adolescent #1

When discussing changes, the adolescents mentioned but were less focused on changes in the frequency of NSSI (although most of them had decreased their frequency of NSSI from before treatment to the time of the interview). Reasons for decreased or ceased NSSI described by adolescents were that they felt less trapped and dared to express their emotions and that they had other strategies to handle impulses/emotions now. Moreover, some adolescents reported that they did not experience positive changes until after several weeks after treatment termination.

The caregivers experienced similar improvements in adolescents. In addition, the caregivers observed that adolescents were more present in the family and forgiving toward oneself and easier to talk to and seemed happier. The caregivers perceived their own improvements as increased self-awareness and emotional awareness—being more aware of their adolescents’ mood—and increased self-efficacy in handling possible setbacks. Moreover, they mentioned that they were teaching the skills to other people around them.

## Discussion

### Principal Findings

This study conveys the experiences of online ERITA for those with NSSI and their caregivers. The main findings were that online ERITA is experienced as offering several means of support (eg therapist, the mobile app) and that the caregiver involvement in this format is acceptable and beneficial. The perceived self-responsibility in online ERITA had both positive (ie, sense of empowerment) and negative aspects (ie, increased distress) connected to it. Several different skills seem to facilitate emotion regulation ability, and having access to such skills could hinder NSSI. Finally, decreased emotion regulation difficulties seemed to be an important treatment outcome for adolescents.

Interestingly, although several treatment effects were described, the most important changes identified by adolescents were improvements in emotion regulation and how that affected functions in daily life. This may not be surprising, given that NSSI per se is seldom the main concern for the individual but rather the emotional distress triggering NSSI [52]. Access to emotion regulation skills was mentioned as a reason for decreased NSSI, illustrating the potential mediating role that emotion regulation has in decreasing NSSI [30,36,53]. Furthermore, the results are in line with those of previous studies [24,30,36], indicating that interventions that are designed specifically for NSSI and target emotion regulation difficulties can affect other symptoms and the overall function. This is expected, as emotion regulation deficits seem to be important to a wide range of psychopathologies [54]. Altogether, these results highlight the importance of measuring several outcomes when evaluating treatment for NSSI to capture the processes through which the treatment works and the changes that are connected to decreased distress and increased function.

Our results indicate that adolescents used several different skills to regulate their emotions. Given that several different subscales of difficulties in emotion regulation have been strongly connected to NSSI (eg, nonacceptance of emotional responses, impulse control difficulties, and difficulties engaging goal-directed behavior) [23], several different emotion regulation skills could be useful to target these different difficulties. Therefore, it does not need to be concerning that no single emotion regulation skill was identified as of certain importance from the interviews with adolecents. Investigating whether it is possible to predict what skills the individual would benefit the most from, based on reports of difficulties in different emotion regulation subscales, could be an important next step to identify potent skills and components and further tailor the treatment to the individual. Online treatment could provide a useful framework for dismantling studies (eg, testing the effectiveness of a specific treatment component) [55] as treatment content could be controlled and modules can be easily added or removed.

In this study, therapist support was perceived as essential and therapeutic alliance was continually developed through the online format. The question has been raised if the online format can facilitate therapeutic alliance when treating self-injury [37]. Our results indicate that the online format facilitated self-disclosure, in line with the experiences of other pediatric clinical groups who have received online treatment [38]. In general, our results validate the importance of therapeutic alliance for this target group [26], which seems to be facilitated by both online, telephonic, and face-to-face contact in this context. The availability of frequent and flexible online and telephonic contact was important for the participants, in line with previous findings [25,27].

The importance of family involvement for this population [25,40] was also underscored from the results of this study. Involving caregivers in a parallel online course was highly appreciated by the caregivers and experienced as positive, or at least not negative, by the adolescents. Involving family members in this online format could be a valuable addition to traditional face-to-face family sessions. The importance of providing a sufficient dose of caregiver or family component has been emphasized [25], and the online format could be a time-efficient method for the therapist to increase such dose. Conversely, face-to-face meetings, as a complement to the online treatment, were requested by caregivers in this study, implying the importance of flexibility in format. Moreover, caregivers’ own skills training in emotion regulation was appreciated and helpful, and this is important as youth NSSI could affect the well-being of caregivers negatively [56].

Several advantages of the supplementary mobile app were described by the adolescents, both as an easy and flexible way to engage in treatment and a means of support. These results, together with the previous promising results on the utility of mobile apps for self-injury [39], indicate the usefulness of supplementary add-on formats for both online and face-to-face treatments. However, not all adolescents used the mobile app, and the reasons mentioned involved not needing it and technical issues. The technical issues are unfortunate but are difficult to avoid in complex apps. The report from some of the adolescents that the technical issues prevented them from using the app underline the need for thorough pretesting of the app and also conducting pilot trials, such as this study, before the app is used in a large-scale clinical trial or implemented within health care. Nevertheless, whenever an app is used, continuous efforts should be made to detect and handle any issues because technical problems could have negative consequences on treatment credibility, adherence, and effectiveness.

Furthermore, difficulties in fulfilling assignments and adhering to treatment [57] and feeling worse on occasions [38] have been stated as possible negative effects of online treatment before. Such distress can be challenging for individuals with NSSI, as difficulties in regulating emotions are especially prominent within this group [23]. Detecting participants experiencing distress because of the self-responsibility inherent in the online format and offer support is therefore important. The findings could be interpreted as a need for more individual tailoring in online treatments for NSSI to facilitate successful treatment outcomes and identify patients in need of adaptions or potentially more extensive treatment first hand. This means of treatment may not be suitable for the most severely affected; although online treatment offers frequent contact that could facilitate detection of deterioration in some cases, there may be dimensions of a face-to-face contact (eg, nonverbal communication) that are lost in online treatment, which could possibly make it more difficult to detect deterioration in other cases. Nevertheless, online treatment could serve important functions; the results from this study indicate that some prefer online contact, and a third of the adolescents in this study (and a fifth in the overall pilot trial [36]) identified as nonbinary, which could indicate that the online format may facilitate treatment-seeking among gender minority groups.

### Strengths and Limitations

Although our study provides important information on how skills training, caregiver involvement, and the online format can be experienced, it is not without limitations. Irrespective of sample size, sampling from a single pilot study limits the transferability of the results [58]. Nevertheless, by including adolescents of different ages, genders, and NSSI frequencies, we could collect rich data, hopefully making the findings transferable in the sense that they speak for more than the individuals interviewed. However, although we strived for variability in the sample, we have no adolescents who identified themselves as male, and none of them were aged 13 years. Moreover, some families were interviewed months after ending the treatment, possibly introducing recall bias but also introducing the possibility of capturing late effects.

### Future Work

Our results indicate the potential development of online ERITA. Primarily, it is important to systematically detect those at risk of distress because of the self-responsibility inherent in the online format. As online treatment generally offers structured treatment content, questions regarding treatment engagement and feelings of increased distress can easily be incorporated into the treatment modules. Accordingly, modifying and evaluating the efficacy of online ERITA is warranted, and a randomized controlled trial is currently being conducted and is registered at ClinicalTrials.gov (trial number: NCT03353961).

If online ERITA is proven to be efficacious, steps have already been taken to prepare for a large-scale implementation trial within Swedish child and adolescent mental health services. In this phase, we will not only monitor treatment effectiveness and potential adverse events but also study patient as well as caregiver experiences and evaluate the intervention from an organizational perspective.

### Conclusions

The findings from this qualitative study exploring the experiences of online ERITA for those with NSSI and their caregivers provide some important insights. First, decreased emotion regulation difficulties were an important outcome for adolescents, implying the importance of targeting emotion regulation and measuring several outcomes when evaluating treatment for NSSI. In addition, several different skills seem to facilitate emotion regulation ability and having access to such skills could hinder NSSI. Second, involving caregivers through a parallel online course seems to be an accepted and beneficial format to deliver family interventions for this patient group. Third, it is possible to learn and practice skills training in online format, with several means of support, from both the mobile app and the therapist. Finally, although online treatment could be empowering, there is a risk of distress because of the self-responsibility inherent in the online format that needs to be addressed.

## Appendix

Multimedia Appendix 1 Interview guide for adolescents.

Multimedia Appendix 2 Interview guide for caregivers.

## Abbreviations

DBT: dialectical behavior therapy
ERGT: emotion regulation group therapy
ERITA: emotion regulation individual therapy for adolescents
NSSI: nonsuicidal self-injury
