# Supplementary material for: Experiences of an Online Treatment for Adolescents With Nonsuicidal Self-injury and Their Caregivers: Qualitative Study
Source: JMIR Form Res. 2021 Jul 23;5(7):e17910. doi: 10.2196/17910 (PMC8367103; doi:10.2196/17910)
Supplement: Multimedia Appendix 1 [file formative_v5i7e17910_app1.pdf]

Interview Guide for Adolescents

Before the interview: Present yourself and explain the aim of the interview. Inform the participant that the interview will be recorded and how the audio file will be processed and stored. Enlighten the participant that information will be presented anonymously. Inform the participant that he/she has every right to terminate the interview at any time, without stating a specific reason. Notify the participant on the estimated time of the interview. Ask the participant to give written and/or oral (adolescents < 15 years old consent only orally) consent. Before the interview starts: ask if the participant has any questions.

Area of interest	Fixed question	Examples of supplementary questions
Online treatment	Could you tell me about your experience of receiving treatment online? How was it?	• How did you work with the treatment?<br>• How did you use the mobile app?<br>• How did you experience the contact with your therapist?
Content of online ERITA	How did you experience the content/material on the internet platform?	• What was the most helpful part of the treatment?<br>• What was less helpful?<br>• What changes could be done to the treatment for it to better suit you?<br>• How did (or did not) the treatment match your expectations?
Involvement of caregiver(s)	How were you affected by the fact that your caregiver (s) had their own material? How did you experience your caregiver(s) involvement?	• How has the treatment affected the relationship between you and your caregiver?

Is there anything else we have not talked about that you want to tell me about?

After ending the interview: ask how it felt to participate in the interview.
