# Supplementary material for: Experiences of an Online Treatment for Adolescents With Nonsuicidal Self-injury and Their Caregivers: Qualitative Study
Source: JMIR Form Res. 2021 Jul 23;5(7):e17910. doi: 10.2196/17910 (PMC8367103; doi:10.2196/17910)
Supplement: Multimedia Appendix 2 [file formative_v5i7e17910_app2.pdf]

Interview Guide for Caregivers

Before the interview: Present yourself and explain the aim of the interview. Inform the participant that the interview will be recorded and how the audio file will be processed and stored. Enlighten the participant that information will be presented anonymously. Inform the participant that he/she has every right to terminate the interview at any time, without stating a specific reason. Notify the participant on the estimated time of the interview. Ask the participant to give oral and written consent. Before the interview starts: ask if the participant has any questions.

Area of interest	Fixed question	Examples of supplementary questions
Online treatment	Could you tell me about your experience of receiving your course online? How was it?	• How did you work with the course?<br>• How did you experience the contact with your therapist?
Content of online ERITA	How did you experience the content/material on the internet platform?	• What was the most helpful part of the course?<br>• What was less helpful?<br>• How could the course be improved to suit you better?<br>• How did (or did not) the course match your expectations?
Relationship with the adolescent	How has online ERITA affected your adolescent and your relationship?	• How do you notice a change (if there is one)?<br>• What in the treatment do you think have contributed to such change?

Is there anything else we have not talked about that you want to tell me about?

After ending the interview: ask how it felt to participate in the interview.
